# Can peritoneal dialysis be continued, as opposed to switching to HD, in end-stage renal disease patients with acute coronary syndrome with pulmonary edema?

**DOI:** 10.1080/0886022X.2025.2534502

**Published:** 2025-07-29

**Authors:** Yahn-Bor Chern, Yu-Ling Lin, Hsi-Hao Wang, Shih-Yuan Hung, Min-Yu Chang, Li-Chun Ho, Ching-Fang Wu, Hung-Hsiang Liou, Yi-Che Lee

**Affiliations:** a Division of Nephrology, Department of Internal Medicine, Yuan’s General Hospital, Kaohsiung, Taiwan; b Division of Nephrology, Department of Internal Medicine, Hsinchu Cathay General Hospital, Hsinchu, Taiwan; c Division of Nephrology, Department of Internal Medicine, Cathay General Hospital, Taipei, Taiwan; d School of Medicine for International Students, College of Medicine, I-Shou University, Kaohsiung, Taiwan; e Division of Nephrology, Department of Internal Medicine, E-DA Hospital, Kaohsiung, Taiwan; f Division of Nephrology, Department of Internal Medicine, Hsin-Jen Hospital, New Taipei City, Taiwan

**Keywords:** Acute coronary syndrome, chronic kidney disease, hemodialysis, end-stage kidney disease, peritoneal dialysis, pulmonary edema

## Abstract

**Background:**

Managing acute coronary syndrome (ACS) with pulmonary edema is challenging in end-stage kidney disease (ESKD) patients on dialysis. While hemodialysis (HD) is often chosen for rapid fluid removal, peritoneal dialysis (PD) may better preserve hemodynamic stability in patients prone to circulatory compromise. This study evaluated whether continuing PD in patients who develop ACS and pulmonary edema while already on PD is feasible for improving oxygenation and fluid management without switching to HD.

**Methods:**

This retrospective single-center study reviewed 13 PD patients who experienced 15 ACS episodes complicated by pulmonary edema. Data collected included demographics, comorbidities, Killip classification, PD regimen modifications, and outcomes. Adjustments to PD prescriptions and their effectiveness were assessed.

**Results:**

Among the 15 episodes, 11 (73.3%) were successfully managed with PD alone, while 4 (26.7%) required temporary HD due to insufficient fluid removal. Most cases were Killip Class II (20%) or III (73.3%). The average ICU stay was 4 days, and in-hospital mortality was 20%.

**Conclusions:**

Continuation of PD in patients who develop ACS accompanied by pulmonary edema appears feasible in most cases, provided that PD prescriptions are carefully individualized. Switching to HD is not invariably required, but thoughtful patient selection and close monitoring remain essential to optimize clinical outcomes.

## Introduction

Acute coronary syndrome (ACS) with pulmonary edema creates significant obstacles to effective fluid management, especially in patients undergoing dialysis [1]. Traditionally, hemodialysis (HD) has been the treatment of choice in these conditions because of its ability to rapidly remove excess fluid and manage acute volume overload. However, in these cases, rapid fluid transfer can lead to hemodynamic instability and arrhythmias, exacerbate myocardial strain, and accelerate intradialytic hypotension, especially in patients with preexisting hypotension or vascular disease [2–6].

In contrast, peritoneal dialysis (PD) provides gradual and continuous fluid removal and may have significant advantages in reducing the risk of sudden changes in blood volume and pressure, particularly in critically ill patients [7,8]. Evidence from several studies indicates that patients with heart failure undergoing PD experience fewer blood pressure fluctuations and a lower rate of intradialytic hypotension than those treated with HD. [7,9].

In clinical practice, there has been controversy whether to continue PD or transiently convert to HD in patients who initially received PD and subsequently developed ACS complicated by pulmonary edema. Given these considerations, this study aimed to evaluate the clinical feasibility of continuing PD without converting to HD in patients with ACS complicated by pulmonary edema. By retrospectively analyzing the clinical outcomes of 13 patients treated with PD during episodes of acute myocardial infarction (a total of 15 events), we sought to determine whether PD provides adequate fluid management for this acute condition.

## Method

This was a retrospective, single-center descriptive analysis of patients with end-stage kidney disease (ESKD) undergoing PD who developed ACS with accompanying pulmonary fluid overload.

This study includes 13 patients who were undergoing PD and subsequently experienced ACS incidents. These patients were treated at the Taiwan E-DA hospital between 2015 and 2021. Inclusion criteria were as follows: ESKD patients undergoing PD > 3 months with confirmed ACS (including STEMI, unstable angina, or NSTEMI) based on clinical presentation, electrocardiogram, and elevated cardiac biomarker (troponin I). In addition, these patients developed pulmonary edema during the ACS episode, as confirmed by radiographic imaging and clinical evaluation. All patients received PD as their primary form of dialysis before the event.

Exclusion criteria included patients with severe cardiac complications requiring immediate mechanical circulatory support (mainly intra-aortic balloon counterpulsation and extracorporeal membrane oxygenation) and patients with severe contraindications such as peritonitis or abdominal surgery to continue PD. In addition, patients without evidence of pulmonary fluid overload were excluded from this study.

Data were retrospectively collected from electronic medical records. The following variables were recorded—demographics including age, sex, body mass index, and duration of PD before the ACS event; comorbidities such as presence of diabetes mellitus, hypertension, dyslipidemia, chronic obstructive pulmonary disease, and cerebrovascular accident; details of ACS (STEMI, unstable angina or NSTEMI), Killip classification [10], peak troponin I levels, and echocardiographic findings (including left ventricular ejection fraction); PD prescriptions (pre-ACS PD prescriptions: fluid type, volume, exchange frequency). Moreover, any adjustments made to the PD regimen during the first 3 days post-ACS were noted (e.g. changes in the type of dialysate, dialysate concentration, or dwell time to enhance fluid removal).

The primary clinical outcomes included intensive care unit stay duration, resolution of pulmonary edema, and whether the patient required transition to HD owing to inadequate fluid management by PD. Mortality rates and discharge status were also recorded.

Resolution of pulmonary edema was primarily determined based on improvements in chest radiography (CXR) findings and oxygen saturation levels. Radiographic improvement was assessed by attending clinicians in conjunction with radiology reports, and oxygenation status was monitored using peripheral oxygen saturation (SpO_2_) measurements.

Descriptive statistical analyses were used to summarize the data. Key trends and patterns observed in patient outcomes were highlighted, and individual cases that demonstrated either successful or unsuccessful PD management during an ACS episode were analyzed in greater detail.

## Results

This study included 13 patients with 15 episodes of ACS while undergoing PD (Table 1). The mean age of the patients across the 15 episodes was 59.6 years (range, 34–78 years), and 53.3% were male. The median time of PD before ACS event was 41.1 months. Comorbidities were common, with 100% of patients diagnosed with hypertension; 84.61%, diabetes mellitus; 92.31%, dyslipidemia. Several patients had a history of cerebrovascular accident or peripheral arterial occlusive disease.

**Table 1.** Baseline data of peritoneal dialysis patients.

No. of event	Date	Patient No.	Gender	Age at event onset	BMI (kg/m^2^)	PD duration (month)	Residual urine (mL/day)	PET*	Hb (g/dl)	C/T ratio (%)	Comorbidity
1	2015/03/06	#1	Male	78	21.7	74	0	HA (0.77)	9.7	38	HTN, dyslipidemia, ischemic stroke
2	2015/05/21	#2	Male	34	23.5	29	900	LA (0.6)	7.6	46	DM, HTN, dyslipidemia
3	2015/11/29	#3	female	57	31.4	96	0	LA (0.64)	9.2	65	DM, HTN, dyslipidemia
4	2016/05/16	#4	Male	51	22.5	83	0	HA (0.74)	10.1	58	DM, HTN, dyslipidemia, PAOD
5	2016/08/03	#5	Female	63	30.5	31	0	HA (0.69)	10.4	61	DM, HTN, dyslipidemia
6	2016/08/30	#6	Male	60	22.8	15	0	LA (0.63)	10.3	54	DM, HTN, dyslipidemia, ischemic stroke
7	2017/01/20	#7	Male	65	25.6	3	900	H (0.82)	5.5	55	DM, HTN, dyslipidemia, ischemic stroke
8	2017/03/18	#8	Female	54	24.0	31	0	LA (0.63)	8.5	53	DM, HTN, dyslipidemia
9	2017/11/15	#9	Male	53	22.0	29	200	LA (0.61)	7.8	55	DM, HTN, PAOD
10	2018/05/15	#10	Male	70	24.0	36	0	HA (0.75)	6.8	60	DM, HTN, dyslipidemia
11	2018/11/04	#11	Female	61	26.3	20	250	HA (0.66)	7.6	58	DM, HTN, dyslipidemia
12	2019/05/06	#8	Female	56	19.8	57	0	LA (0.55)	9.4	59	DM, HTN, dyslipidemia, PAOD
13	2020/07/09	#12	Male	69	29.2	49	40	HA (0.67)	11.4	65	DM, HTN, dyslipidemia, ischemic stroke
14	2021/08/17	#13	Female	62	22.4	30	710	HA (0.76)	9.8	56	DM, HTN, dyslipidemia, PAOD
15	2021/12/08	#13	Female	62	24.1	34	710	HA (0.76)	10.4	58	DM, HTN, dyslipidemia, PAOD

BMI denotes body mass index, PD peritoneal dialysis, PET peritoneal equilibration test, Hb hemoglobin, C/T ratio cardiothoracic ratio, DM diabetes mellitus, HTN hypertension, PAOD peripheral arterial occlusive disease.

*PET: 4-h D/P creatinine (dialysate-to-plasma creatinine ratio).

Of these 15 events, 80% were diagnosed as non-ST elevation myocardial infarction (NSTEMI) and 20% as ST-elevation myocardial infarction (STEMI). With respect to Killip classification, 20% of the events were categorized as Killip II, 73.3% as Killip III, and 6.7% as Killip IV.

The central goal of the study was to examine whether PD could be effectively continued during ACS episodes without switching to HD. Of the 15 episodes, 73.3% (*n* = 11) of patients were able to continue PD treatment without transitioning to HD (Table 2). This included one patient who temporarily suspended dialysis for 1 day owing to severe hemodynamic instability. All patients who continued PD had their dialysis prescriptions adjusted to enhance fluid removal, typically involving an increase in the hypertonic dextrose dialysate concentration, increased use of Icodextrin, shortened dwell times, and increased exchange frequency to prevent fluid overload. In 20% of cases (*n* = 3), HD was initiated owing to inadequate fluid management.

**Table 2.** Cardiac conditions, peritoneal dialysis prescriptions# and outcomes.

No. of event	Type of acute coronary syndrome	Killip Classification	Initial APACHE II score	Troponin I (baseline) (ng/mL)	Troponin I (peak) (ng/mL)	Echocardiography LVEF* (VHD)	Pre ACS PD prescription	Post ACS day 1 PD prescription	Post ACS day 2 PD prescription	Post ACS day 3 PD prescription	Urine output (mL/day), ultrafiltration rate (mL/day), BW (kg) post ACS day 1, 2, 3	Inotropic, diuretics, outcome (ICU duration, expire)
1	STEMI	II	24	0.10	33.62	60% (calcified aortic valve area 1.9cm^2^, mild TR)	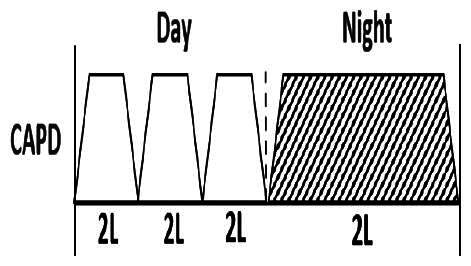	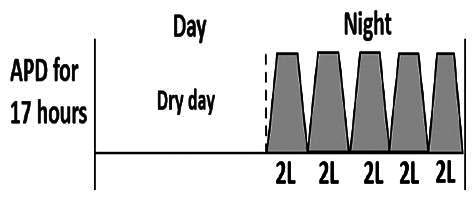	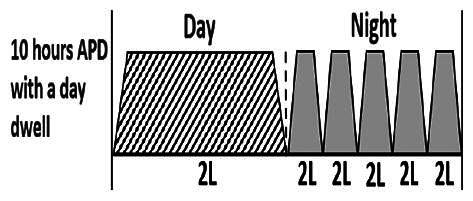	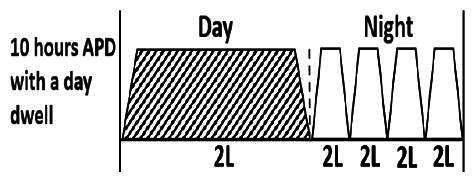	N/A, N/A, N/A 1066, 1074, −168 56.7, 56,5, 56,5	+ - Critical AAD (ICU: 5 days)
2	NSTEMI	III	12	<0.01	23.86	55% (Mod. TR, mild MR)	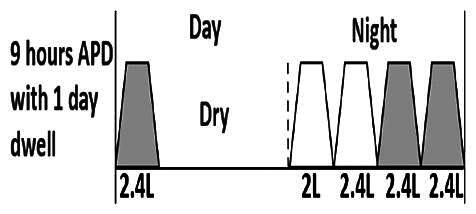	HD	HD	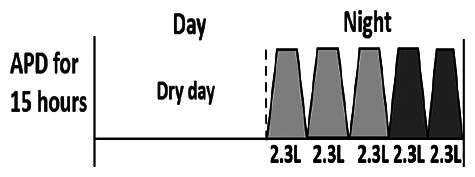	N/A, N/A, N/A 2700, 3000, 1290 70.5, 67.6, 65,2	- - Discharged (ICU: 6)
3	NSTEMI	III	16	0.03	11.43	51% (Mild MR, TR), IE	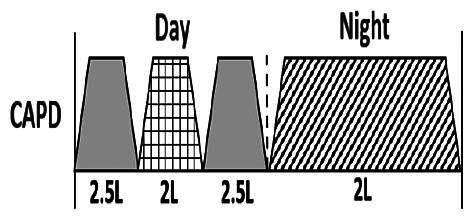	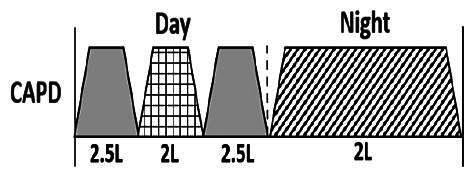	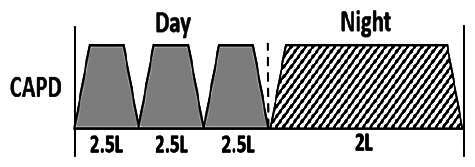	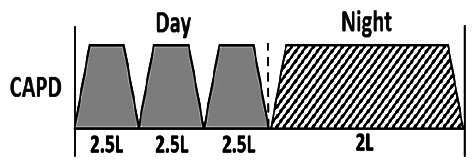	N/A, N/A, N/A 701, 1580, 1479 74.8, 73.9, 73.4	- - Discharged (ICU: 5)
4	NSTEMI	III	13	0.22	21.00	62% (Mild MR, TR and PR)	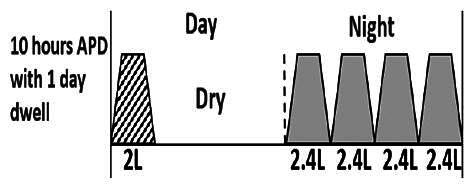	HD	–	HD	0, 0, 0 3400, -, 3000 57.8, 54.7, 54.4	- - Discharged (ICU: 6)
5	NSTEMI	III	18	N/A	16.61	45% (mild TR, AR, mod. MR)	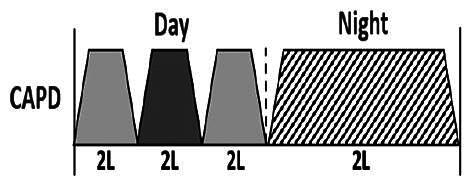	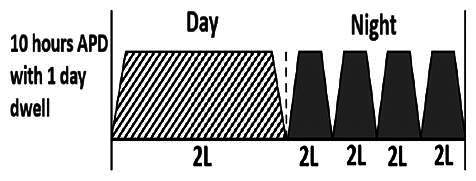	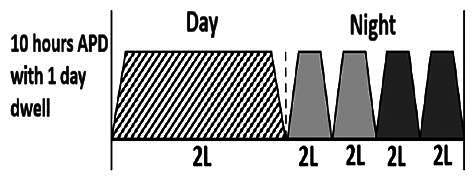	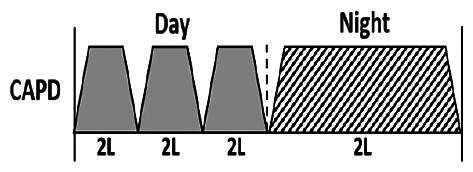	110, 0, 0 3087, 2321, 1800 66.8, 66.3, 65.1	- - Discharged (ICU: 3)
6	STEMI	II	31	N/A	110.22	34% (Mild MR, TR)	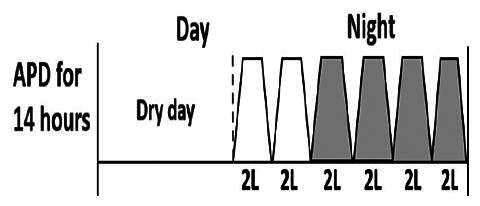	Hold PD (unstable hemodynamics)	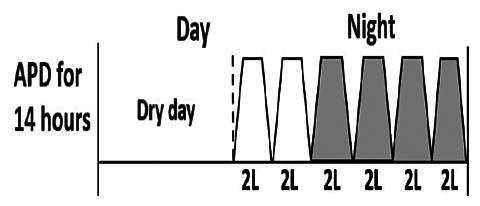	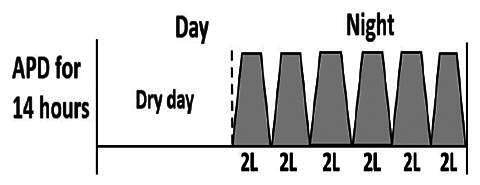	0, 0, N/A -, 873, 782 55.0, 55.8, 55.8	+ - Critical AAD (ICU: 3)
7	NSTEMI	III	21	0.03	0.99	68% (Mild MR, TR)	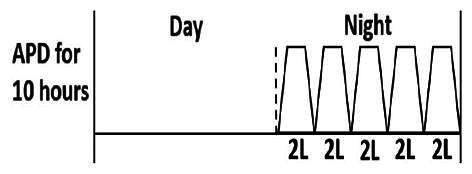	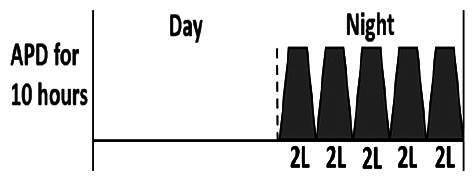	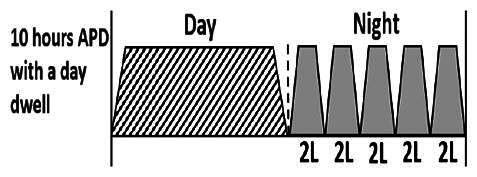	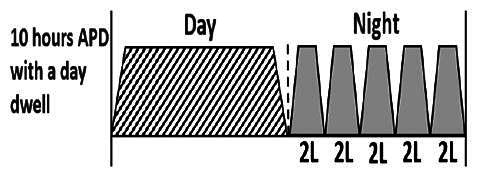	630, 600, 450 2560, 1729, 1035 72.3, 73.3, 73.1	- - Discharged (ICU: 5)
8	NSTEMI	III	N/A	0.04	1.43	68% (mild MR, PR, TR)	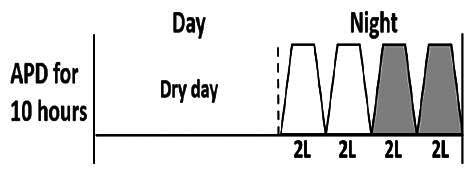	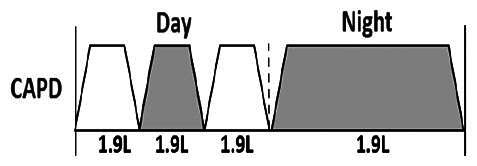	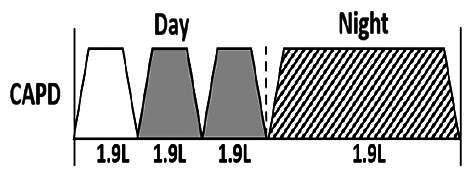	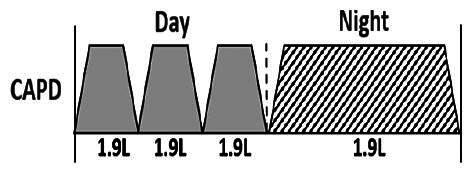	0, 0, 0 800, 400, 800 57.9, 57.4, 55.9	- - Discharged (ICU: 0)
9	NSTEMI	III	21	0.15	21.77	45% (mod MR, AR and TR)	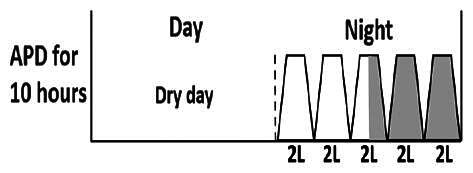	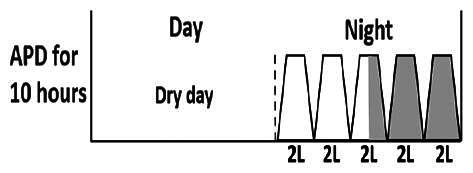	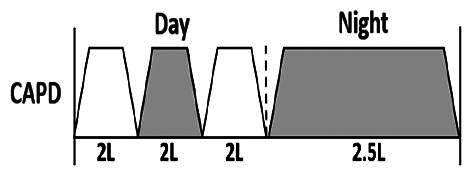	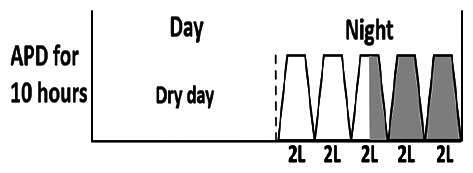	0, 200, N/A loss, 800, loss 61.5, N/A, 64.6	- - Discharged (ICU: 2)
10	NSTEMI	III	24	0.01	1.23	37% (mod MR, TR)	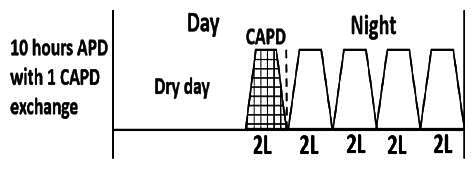	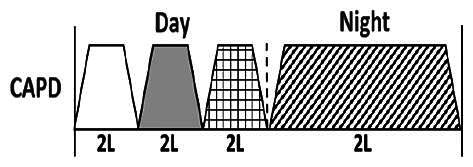	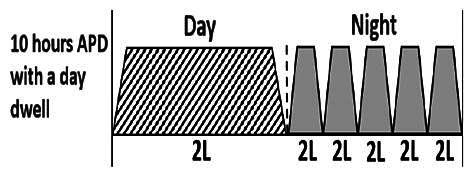	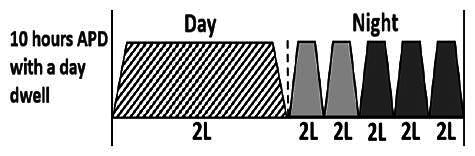	0, 0, 0 loss, 492, 383 67.9, 70.1, 70.6	- - Discharged (ICU: 8)
11	NSTEMI	II	19	0.02	1.31	63% (mild AR and MR)	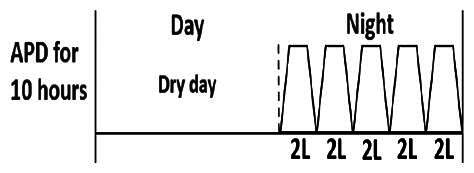	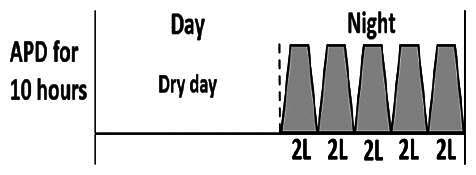	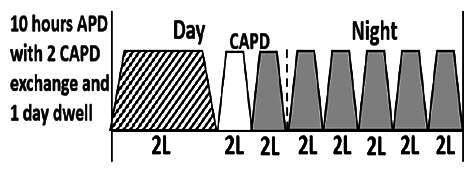	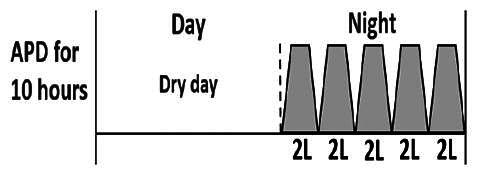	60, 280, 400 1099, 1526, 1245 53.8, N/A, 51.5	- +^^^ Discharged (ICU: 4)
12	NSTEMI	III	16	0.04	26.72	33% (mod MR, mild TR)	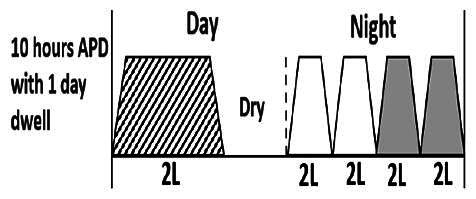	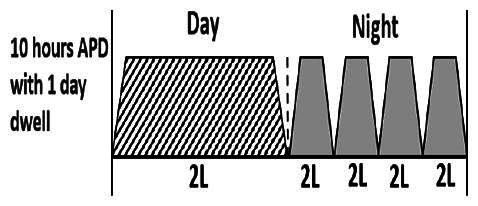	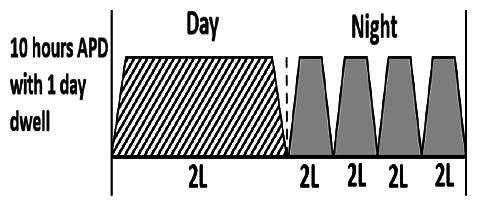	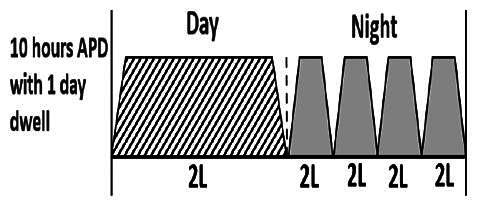	0, 0, 0 1735, 2194, loss 49.6, 51.2, 49.9	- - Discharged (ICU: 3 + 7)
13	STEMI	IV	25	N/A	31.82	N/A	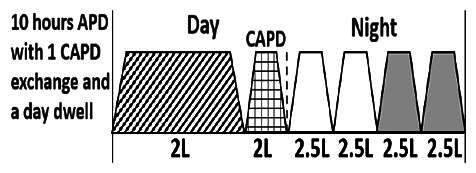	Death	Death	Death	N/A	+ - Mortality (ICU: 1)
14	NSTEMI	III	N/A	0.01	2.08	60% (mod. MR, mild TR)	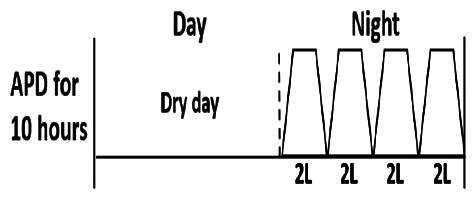	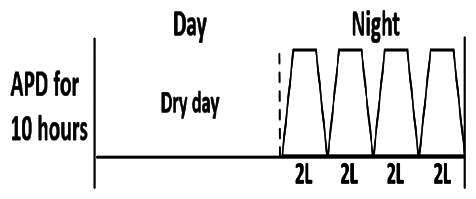	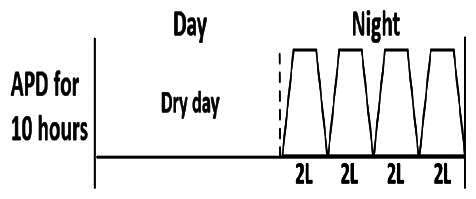	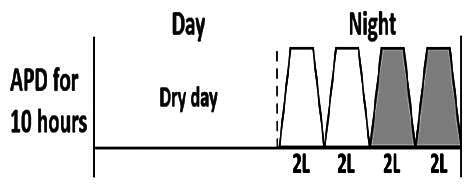	N/A, N/A, N/A 822, 632, 1351 N/A, 54, N/A	- - Transfer for CABG (ICU: 0)
15	NSTEMI	III	16	0.01	22.17	55% (mild MR and TR)	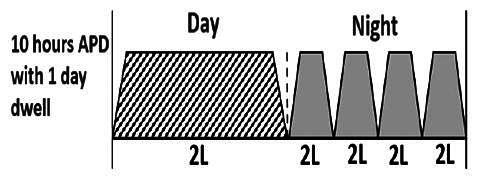	HD	HD	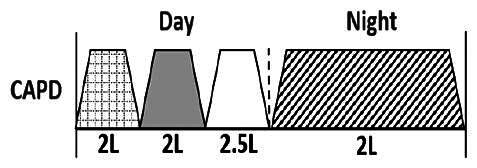	350, 0, 0 3000, 2000, 1300 57.0, 56.8, 58	+ - Discharged (ICU: 3)

* Peritoneal dialysis prescriptions are illustrated below.

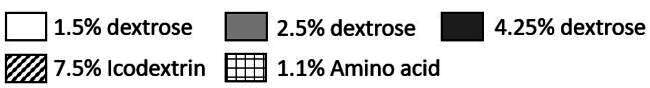
.

*The left ventricular ejection fraction was calculated using the Teichholz method from M-mode echocardiography measurements.

APACHE denotes Acute Physiology and Chronic Health Evaluation, PD peritoneal dialysis, HD hemodialysis, ACS acute coronary syndrome, ICU intensive care unit, STEMI ST elevation myocardial infarction, NSTEMI non-ST elevation myocardial infarction, AAD discharge against medical advice, MR mitral regurgitation, TR tricuspid regurgitation, AR aortic regurgitation, PR pulmonary regurgitation, mod moderate, ACS acute coronary syndrome, CABG coronary artery bypass grafting.

^^^Furosemide 40 mg administered intravenously every 12 h.

All patients were admitted to the ICU with a mean ICU stay of 4.0 days (range, 0–10 days), and an in-hospital mortality rate of 20% (*n* = 3). Two of the patients who died had concomitant septic shock during hospitalization, while the remaining one patient died of profound cardiogenic shock within 1 day of being diagnosed with myocardial infarction. One patient was transferred to another medical center for coronary artery bypass grafting because of personal preference, and the remaining surviving patients were discharged from the hospital after stabilization of their cardiac and fluid statuses.

To further explore potential differences between patients who continued PD and those who were switched to HD, we conducted a subgroup comparison based on dialysis modality. Table 3 summarizes the baseline demographic and clinical characteristics of both groups. Due to the small sample size, particularly in the HD group (*N* = 3), several continuous variables were reported using observed ranges rather than means or medians to better reflect the variability in individual clinical characteristics. However, these observations should be interpreted with caution given the limited sample size and the absence of statistical testing.

**Table 3.** Baseline demographic characteristics of patients who continued peritoneal dialysis versus those who switched to hemodialysis.

Variables	Switched to HD group (*N* = 3)	Continued PD group (*N* = 11)
Male	2 (66.7%)	5 (45.4%)
Age at event onset	34–62	53–78
BMI (kg/m^2^)	22.5–24.1	19.8–31.4
PD duration (month)	29–83	3–96
Residual urine (mL/day)	0–900	0–900
PET type	2 HA/1 LA	1 H/5 HA/5 LA
PET (4-hour D/P creatinine)	0.6–0.76	0.55–0.82
Comorbidity, n (%)		
DM	3 (100%)	10 (90.9%)
Hypertension	3 (100%)	11 (100%)
Dyslipidemia	3 (100%)	10 (90.9%)
Ischemic stroke	0 (0%)	3 (27.2%)
PAOD	2 (66.6%)	3 (27.2%)
Hb (g/dl)	7.6–10.4	5.5–10.4
C/T ratio (%)	46–58	38–65
Type of acute coronary syndrome	All NSTEMI	2 STEMI/9 NSTEMI
Killip Classification	All class III	3 class II/8 class III
Initial APACHE II score	12–16	16–31
Troponin I (peak) (ng/mL)	21.01–23.86	0.99–110.22
Echocardiography LVEF (VHD)	50–62	33–68
Ultrafiltration rate (mL/day)	HD: 2000–3400/per session PD: 1290–1300 (2 cases)*	−168–3087

*In two patients from the switched to HD group, peritoneal dialysis was resumed on Day 3 following clinical improvement, and HD was subsequently discontinued.

HD denotes hemodialysis, PD peritoneal dialysis, BMI body mass index, PET peritoneal equilibration test, D/P creatinine dialysate-to-plasma creatinine ratio, DM diabetes mellitus, PAOD peripheral arterial occlusive disease, Hb hemoglobin, C/T ratio cardiothoracic ratio, NSTEMI non–ST-elevation myocardial infarction, STEMI ST-elevation myocardial infarction, APACHE II Acute Physiology and Chronic Health Evaluation II, LVEF left ventricular ejection fraction, and VHD valvular heart disease.

## Discussion

This retrospective study, involving 13 patients and 15 episodes of ACS complicated by pulmonary edema identified clinically or radiographically during peritoneal dialysis, offers important experience to inform feasibility and clinical results. The main result of this study is that 73.33% of patients were treated on PD alone and there was no need for escalation to HD for the reasons related to fluid therapy. Although HD is often considered the modality of choice for acute decompensated heart failure owing to its capacity for rapid fluid removal, this study demonstrated that through adjustments to PD prescriptions, adequate fluid removal can be achieved in the majority of patients. Most patients were successfully managed with PD without the need to escalate to HD, suggesting that with appropriate prescription modifications, PD can be a feasible option for fluid management in such acute scenarios.

Although HD is widely recognized for its ability to achieve rapid ultrafiltration, PD may reduce the risk of complications associated with abrupt fluid shifts, which is particularly beneficial in patients with preexisting cardiovascular disease [11,12]. In these settings, PD offers potential clinical benefit, especially in patients with chronic heart failure or hemodynamic instability. Previous studies have shown that PD improves outcomes in patients with refractory heart failure, improves New York Heart Association functional class, increases left ventricular ejection fraction, and reduces body weight and length of stay [13–16]. In addition, in critically ill patients with acute kidney injury, a single-center randomized controlled study comparing CVVHDF to PD found no significant differences in the ultrafiltration volumes achieved over the first week [17]. In particular, PD was associated with significant reductions in blood urea nitrogen and potassium levels, suggesting that PD is not only comparable but potentially superior in managing fluid and electrolyte balance in such critical situations. These findings support PD as a safe and effective option for the treatment of ACS complicated by pulmonary edema.

This study highlighted most cases in which PD was successfully used to manage fluid overload in ACS with episodes of pulmonary edema. These cases demonstrate that individualized peritoneal dialysis regimens, such as increased dialysate concentration, icodextrin use, and more frequent exchanges, can effectively control pulmonary edema while maintaining cardiac stability. Notably, there are two important considerations. First, caution is warranted when increasing the number of PD cycles within a limited time frame, as shorter dwell times may induce the phenomenon of sodium sieving—where water is rapidly removed through aquaporins while sodium removal is delayed—potentially resulting in sodium retention and suboptimal fluid clearance. This physiological limitation should be taken into account when optimizing PD prescriptions in volume-overloaded patients. Second, the use of high-concentration (4.25%) dextrose dialysate was a common strategy to enhance ultrafiltration. However, it is important to be mindful of potential metabolic consequences, especially in diabetic patients, as the high glucose content can increase peritoneal glucose absorption and lead to episodes of hyperglycemia. In contrast, some patients with more severe pulmonary edema (Killip class III or IV) may require more aggressive fluid removal with HD or continuous renal replacement therapy. These findings emphasize the importance of clinical judgment in determining when to continue PD or switch to HD to provide optimal treatment based on the patient’s condition.

This study has several limitations. First, only 13 patients and 15 episodes of ACS were included, which limits the statistical power and generalizability of the findings.

Second, the study was conducted retrospectively at a single center, which may have introduced selection bias and affected the accuracy and external validity of the data.

Third, as a retrospective study without randomization, there is a risk of selection and information bias, further limiting the generalizability of the results.

Fourth, this study focused only on in-hospital outcomes and lacked long-term follow-up data, making it impossible to assess the long-term feasibility and safety of continuing peritoneal dialysis.

Fifth, the adjustment of PD prescriptions and the decision to continue PD or switch to HD were made by different attending physicians based on clinical judgment, which may have introduced variability in management and affected consistency and reproducibility. In addition, we have noted that the absence of standardized protocols for PD prescription adjustments may limit the reproducibility and applicability of our findings, and that future studies should aim to develop and evaluate standardized PD management strategies for patients with ACS and pulmonary edema.

Lastly, the study was based on descriptive statistics, without inferential statistical analyses. As a result, statistical significance between subgroups could not be determined, limiting the strength of the conclusions.

In conclusion, this study indicate that peritoneal dialysis can be safely continued in appropriately selected patients experiencing acute coronary syndrome complicated by pulmonary edema. With appropriate adjustments to the PD regimen, effective fluid management can often be achieved without the need to transition to hemodialysis. Further large-scale, prospective, multicenter studies with randomization and long-term follow-up are needed to validate these findings and to support the development of standardized protocols for adjusting peritoneal dialysis regimens in this population.

## Data Availability

The data used in this study were collected from E-Da Hospital and have been de-identified to protect patient confidentiality. Access to the raw data is restricted due to ethical and privacy considerations, as approved by the hospital’s Institutional Review Board (IRB). However, processed and anonymized datasets may be made available upon reasonable request to the authors with the approval of E-Da Hospital.
